# Knowledge, attitudes and bite prevention practices and estimation of productivity of vector breeding sites using a Habitat Suitability Score (HSS) among households with confirmed dengue in the 2014 outbreak in Dar es Salaam, Tanzania

**DOI:** 10.1371/journal.pntd.0007278

**Published:** 2020-07-02

**Authors:** Daniel Msellemu, Tegemeo Gavana, Hassan Ngonyani, Yeromin P. Mlacha, Prosper Chaki, Sarah J. Moore

**Affiliations:** 1 Environmental Health and Ecological Sciences Department, Ifakara Health Institute, Ifakara, Tanzania; 2 Swiss Tropical and Public Health Institute, Socinstrasse, Basel, Switzerland; 3 University of Basel, Basel, Switzerland; National Center for Atmospheric Research, UNITED STATES

## Abstract

**Background:**

The frequency and magnitude of dengue epidemics has increased dramatically throughout the tropics in the past 40 years due to unplanned urbanization, globalization and lack of effective mosquito control. The commercial capital of Tanzania, Dar es Salaam, is now experiencing regular dengue outbreaks. Three dengue serotypes have been detected in Dar es Salaam (DNV 1, 2 and 3). Without adequate vector monitoring and control, further outbreaks will certainly occur.

**Methods/Findings:**

A case series study followed 97 individuals with confirmed dengue fever (NS1 and/or IgM on rapid diagnostic test and/or PCR positive) to their households in Kinondoni, Dar es Salaam during the 2014 outbreak from a random sample of 202 confirmed cases at Mwananyamala Hospital. Kinondoni wards of Manzese, Mwananyamala, Tandale and Mabibo had the highest number of confirmed cases: 18, 13, 13 and 9 respectively. Individuals were interviewed by questionnaire on dengue prevention practices and houses were inspected for mosquito breeding sites to validate a Habitat Suitability Score (HSS). This is a tool devised to predict the productivity of any potential breeding habitats (PBHs) before the rains begin.

There were 12 /312 positive Aedes breeding habitats. Drums/barrels, flowerpots and tyres were the most common breeding habitats. The HSS correctly identified 9/12 of Aedes breeding habitats. Larviciding is already conducted in urban Tanzania for malaria control and the HSS may be a useful means to train individuals on productive *Aedes aegypti* breeding sites should this program be extended to include dengue control.

The population remains poorly informed about dengue transmission and prevention: 22% of respondents said dengue is spread from one person to another and 60% first heard about dengue when already sick. Less than 20% of respondents used personal protection and >80% thought bednets protected against dengue. Mobile phones were owned by almost all individuals followed up and have the potential of being the prime medium for dissemination of information on dengue prevention.

## Author summary

Dengue fever is a viral infection transmitted by Aedes (Stegomyia) mosquitoes causing a flu-like illness that may develop into severe complications such as dengue haemorrhagic fever and dengue shock syndrome if the patient contracts two viral serotypes concurrently. Three dengue serotypes have been detected in Dar es Salaam (DNV 1, 2 and 3). There is currently no antiviral treatment or vaccine available against dengue so vector control and mosquito bite prevention remain essential to prevent transmission. Due to globalisation and rapid urban expansion, Dar es Salaam is experiencing regular dengue outbreaks. Without adequate vector control and public awareness, these will certainly continue to re-occur. During the dengue outbreak of 2014 we followed up 97 people with confirmed dengue. We went to their homes to ask them about their perception of dengue and to look at the suitability of their homes for vector breeding.

We devised a tool to predict whether *Aedes aegypti* would be present in breeding sites based on the results of other authors in the region. The study proposes the use of HSS, a tool to be used to predict the productivity of Aedes breeding habitats, which correctly predicted the presence of *Aedes aegypti* 75% of the time. The prediction will enable quick management of habitats before the rain begins.

The study interviewed those who had confirmed dengue and identified a large gap in public knowledge of dengue fever: 84% of people interviewed thought bed nets can prevent dengue, 60% of the patients only became aware of dengue while in the hospital with the illness and 22% thought that dengue spreads from person to person. The public need to be aware of dengue prevention and management especially just before the rains. Most respondents owned mobile phones and the use of mobile phone messages might be a suitable means to raise awareness before possible outbreaks.

## Introduction

In 2014, a Type 2 dengue fever outbreak in Tanzania spread to seven regions on the mainland and two regions in Zanzibar [1]. On mainland Tanzania, there were 1,017 confirmed cases from a total of 2,121 suspected cases including 4 deaths. Zanzibar had 1 confirmed case out of 8 suspected cases and no deaths. Ninety-nine per cent (99%) of the cases of the mainland were reported from the following three districts of Dar es Salaam: Kinondoni, Temeke, and Ilala. Of the four fatal cases, 1 had presented with Dengue Haemorrhagic Fever (DHF) and 1 with multiple organ failure [2].

Dengue is currently the most widespread arboviral (insect-transmitted virus) disease of humans. The frequency and magnitude of dengue epidemics have increased dramatically in the past 40 years as both the mosquito vectors and the four dengue virus serotypes have expanded geographically throughout the tropics and subtropics. The principal factors driving the massive expansion of epidemic dengue are 1) unplanned urbanization, 2) globalization and 3) lack of effective mosquito control [3]. The vector of dengue in Tanzania is *Aedes aegypti* a mosquito that is highly adapted to humans [4] and thrives in urban settings. Tanzania’s urban population is rapidly expanding with 30% of the population now living in urban areas [5]. In Dar es Salaam there is widespread unplanned urbanisation without waste management or regular water supply. This results in extensive areas of household waste such as plastic containers and household water storage that provide ample breeding sites for *Ae*. *aegypti* [6]. Currently, 4.3 million people live in Dar es Salaam, which also hosts Tanzania’s largest airport and handles 90% of shipping cargo. Tanzania has strong trade and economic links with many dengue endemic countries in South-east Asia providing a route for the introduction of new serotypes.

The reported incidence of dengue has increased worldwide in recent decades, but little is known about its incidence in Africa. During 1960–2010, a total of 22 countries in Africa reported sporadic cases or outbreaks of dengue [7] and the frequency of reported outbreaks is increasing. The presence of disease and high prevalence of antibodies to dengue virus in available serologic surveys suggest endemic dengue virus infection in many parts of Africa, including Tanzania [8]. Dengue is likely under-recognized and underreported in Africa countries due to a low awareness among healthcare providers, other prevalent febrile illnesses, and lack of diagnostic testing and systematic surveillance. While Dar es Salaam has in the recent past experienced dengue outbreaks, without adequate vector control it is certain that these will re-occur. Three dengue serotypes have now been observed circulating in the country [6, 9] and 22 million people live in areas of Tanzania suitable for transmission of arboviruses [10].

### Dengue control

**Fig 1:**
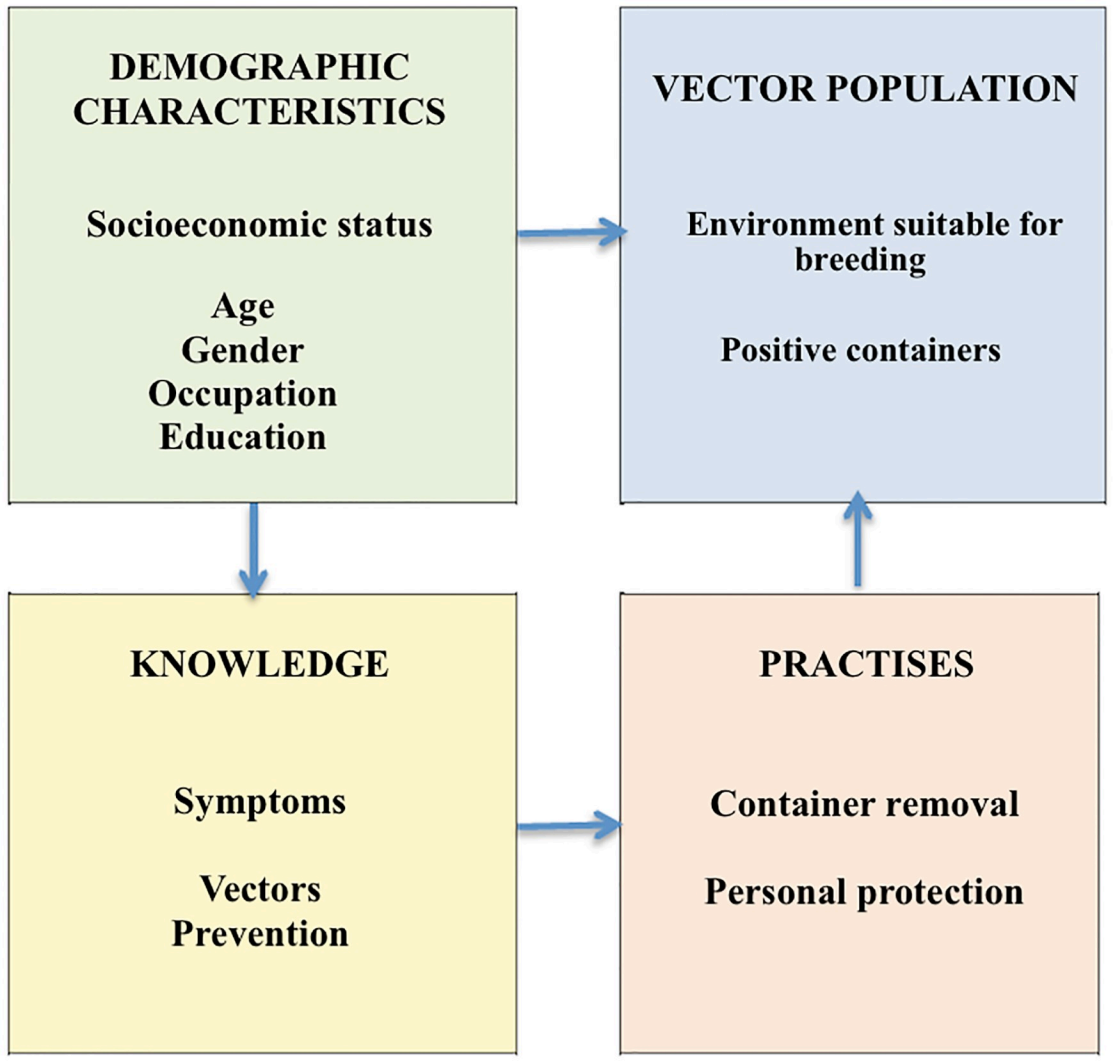
Factors involved in the control or spread of dengue through human and *Aedes aegypti* interaction.

There is currently no antiviral treatment or vaccine against dengue, although a number of vaccines are under development [11]. Thus, prevention and control of dengue virus transmission currently depends entirely on killing the mosquito vectors or preventing mosquito bites [12]. While insecticide treated nets (ITNs) are widespread throughout sub-Saharan Africa, *Ae*. *aegypti* mosquitoes bite during the day and commonly bites outdoors, rendering ITNs ineffective against them. It has been shown that efforts to control *Ae*. *aegypti* are only sustainable through conscious, systematic, proactive and preventive control. Cuba has remained dengue free for 30 years by using a “set of measures directed towards the detection and elimination of possible mosquito breeding places. It hinges on weekly self-directed inspection by families and workers in their homes and workplaces” [13]. This is an example of successful environmental management i.e. changing the environment to prevent vector breeding by destroying, altering, removing or recycling containers that provide potential mosquito-breeding habitats and should be the mainstay of dengue vector control. *Ae*. *aegypti* uses a wide range of confined man-made and natural larval habitats that vary enormously in the number of mosquitoes that they produce [14]. It is therefore most cost and time efficient to target only those habitats that are most productive and therefore epidemiologically more important rather than blanket coverage all types of containers [12]. With this in mind, we followed up Tanzanian patients with confirmed cases of dengue during the 2014 outbreak to characterize possible risk factors for dengue during the dengue fever outbreak in Dar es Salaam “Fig 1”. We visited the homes of confirmed cases and assessed breeding sites to test a tool to predict dengue vector breeding habitat potential during the dry season (habitat suitability-score) that could be used for training larval control staff to prevent epidemics. In addition, we conducted a short questionnaire on dengue patients’ daily activities and travel behaviour around the time of infection to characterise behaviour that might increase the risk of exposure. The epidemiological and entomological characterisation of the dengue outbreak is of high importance to the Tanzanian Government to identify strategies needed for the prevention of future outbreaks.

## Methodology

### Study area and population

**Fig 2:**
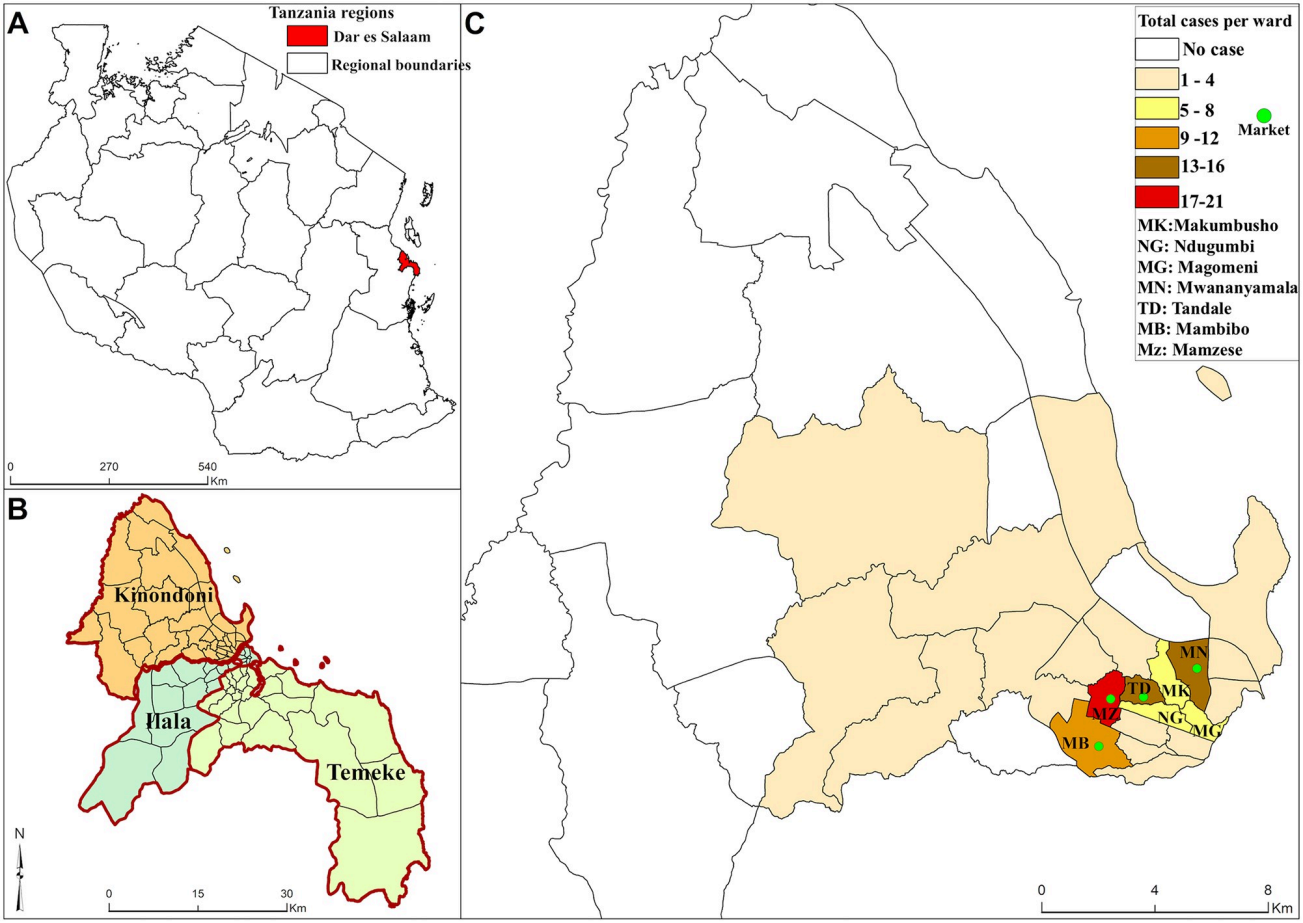
The map of (a) Tanzania, (b) Dar es Salaam, (c) Kinondoni wards with a sample of dengue cases in 2014 (n = 97).

We conducted a case series study that followed up individuals from Kinondoni district with dengue confirmed by NSI antigen or IgM detection by a Rapid Diagnostic Test (RDT) (SD Biolone Dengue DUO) at Mwananyamala Hospital between January and July 2014 [15]. Follow up visits were carried out between 1^st^ and 28^th^ July 2014. Kinondoni is at the northeastern part of Dar es Salaam city “Fig 2” with an area of 531 km^2^, 2,497,940 population and a population density of 1,179 persons per square kilometre [16]. It has an average annual temperature of 25°C and precipitation of 132.9 mm in the rainy season (May–October) [16]. The district has a non-continuous water supply (every 2 days), which leads to widespread water storage, and irregular refuse collection that creates man-made containers that are Potential Breeding Habitat (PBHs) for mosquitoes.

### Study site

The “Etiologies of acute febrile illness among adults attending an outpatient department in Dar es Salaam” ClinicalTrials.gov Identifier: NCT01947075 generated a list of 202 adult Tanzanians with confirmed dengue fever from the out-patients department of Mwananyamala district hospital and in other health facilities in Kinondoni District (Sinza hospital, Magomeni healthcare centre, and Tandale dispensary) in Dar es Salaam. From this list, a selection of 100 participants was obtained by computer-generated randomization without repetition. Out of 100 randomly selected cases we obtained 97 cases with the remaining 3 participants lost to follow up. Only adults of over 18 years of age who were residents of Kinondoni municipality and were sick during the outbreak were interviewed as no children were included in the clinical study, although there were dengue cases among children at that time (Emiliana Athumani pers comm). It should be noted that around 200 cases in non-Tanzanian nationals also occurred but these persons were not followed up as part of this study because they did not receive treatment in Kinondoni Municipality at Mwananyamala hospital [17].

### Data collection

The study collected information on dengue patient history before and after the dengue outbreak. The data collection team visited patients’ homes and upon written informed consent they conducted a short questionnaire on behaviours that increase risks of Aedes mosquito bites and examined the environment looking for breeding sites and then collected larval mosquitoes found in and around the home. The questionnaire (Supplementary file) was developed in English then translated into Kiswahili and piloted by administering it to 3 households to check the ease of understanding. The survey collected information on disease history, outdoor activities, travel in the two weeks preceding their diagnosis, participants’ use of mosquito bite prevention and dengue awareness. Personnel administering the questionnaire did not prompt participants, and were trained to engage in conversation around the topic to gather information without listing or prompting a series of responses. In addition, inside the home and the area around the home was examined for mosquito larvae and photographs were taken of all potential mosquito breeding sites. The study was conducted after the peak of dengue infection due to delays in obtaining ethical approval although the tail end of the epidemic was ongoing.

Twelve Kinondoni municipal health workers were recruited to collect data. They received two weeks of training on informed consent, questionnaires, larval sampling and photography. The health workers were familiar with Kinondoni district as they had continuously been involved in Dar es Salaam Urban Malaria Control Program (UMCP) and are highly experienced in community liaison and larval mosquito sampling.

#### Rainfall data

To establish rainfall patterns and dengue outbreak, rainfall data from two months before the outbreak of December 2013 to July 2014 was obtained from the Tanzania Meteorological Agency (TMA). The rainfall data were compared to dengue fever outbreak records at Mwananyamala Hospital in Kinondoni District. Dengue cases were then analysed against the rainfall data for the duration of the outbreak period. Average rainfall was plotted against numbers of dengue cases confirmed in that month with a two week time-lag between the rainfall and incidence of the cases [18] to account for the time period between rainfall and cases as seen by other authors [19, 20].

### Larval collection

All PBHs both indoors and outdoors, wet or dry were inspected and assessed for the presence or absence of mosquito larvae and pupae. Areas that qualified these criteria were photographed as possible breeding habitat for Aedes mosquitos [21, 22]. Aedes larvae were collected using a larval dipper or pipette from PBHs in and around the dengue patients’ houses to within 100 metres radius or to the closest property (around 30 metres) where houses were crowded. Therefore, a larger area was observed for larger homes. All types of containers were thoroughly checked. To avoid leaving behind Aedes larvae, all larvae of any species found in a PBH were collected. The larvae were then stored at IHI insectary in emergence breeders for later identification of adults.

Aedes lay eggs during the day in dark-coloured containers with wide openings, in shaded locations and prefer water rich in organic materials such as decaying leaves and algae [21, 22] Suitable sites include tree-holes, plant axils, and artificial containers, plant pots and vases, discarded tires rainwater tanks, wells, and metal drums, garbage bins and upturned lids, buckets, cups, glasses, jugs, bowls, dishes, trays, drink cans, food containers and pet bowls [23], while old tyres, water storage containers and vegetation were identified to be the most productive breeding habitat in Dar es Salaam [24].

### Photography and habitat suitability score

A HSS is a proposed tool to be used in assessing Aedes mosquito breeding habitats even before the rains begin. The tool utilises *Aedes aegypti* bionomics reports of Christopher 1960 [22] and represents the sum of points allocated for each feature of a breeding site that makes it conducive to *Ae*. *aegypti* breeding “Table 1”. Photographs of PBHs were taken without zooming to minimize distortion following a standard operating procedure. Photos were given enumeration number linked to the compound and the PBH. Photographs of larval habitats taken during the survey were given HSS based on criteria in “Table 1”. The HSS is scored for each habitat independently with a cumulative score calculated based on each of the features present in “Table 1”. For instance a breeding site with *Ae*. *aegypti* and other mosquitoes present with accumulated clean water that is fully shaded would score 5+3+5+5 = 18 points. A household will get an average suitability score "mean habitat suitability score" based on the sum of all the breeding sites identified. A house with a higher mean habitat suitability score is more likely to be a breeding ground for Aedes mosquitoes and should be targeted for dengue vector control.

**Table 1:** *Aedes aegypti* breeding habitat suitability scores among houses with confirmed dengue cases.

Constituent	Presence	Score
**Larval**	Number of breeding habitat 1/2/3/4/∞	1–10
	*Aedes* aegypti larvae identified	5
	Other mosquito larvae identified	3
**Water**	Accumulated clean water	5
	Water storage jars	5
	Pools of water with vegetation	5
**Shade**	Fully shaded habitats	5
	Partially shaded habitats	3
	Plant axils/flower pots	3
**Environmental**	Scatted containers	5
	Tires	5
	Ability to accumulate water during rain season	5

### Data management

Questionnaire data were double entered into an Epi Info template corresponding to the questionnaire with drop-down lists of legal values and exported into STATA 12. Data were cleaned through checking for data distribution, unusual values and outliers. Thereafter data was uploaded onto the IHI data server for archiving. No data of a personal nature was collected and the households were identified by GPS.

### Data analysis

Data were analysed following a predefined analysis plan by descriptive analysis: cross-tabulation with one-tailed Pearson’s Chi-Square to describe relations between dengue infection, age and gender, travel history, recall of government dengue messages and use of personal protection.

Two steps were used to validate HSS. Firstly, the assessment included all photo-scores and in absence Aedes and other mosquito larvae “Table 1”. Secondly, the photoscore of the same habitats but at this time it includes Aedes larvae scores after laboratory results of hatched larvae. A two-tailed paired t-test was used to assess the means difference between the two samples.

The spatial distribution of dengue cases was mapped by using ArcGIS, version 10.5, CA, USA. Coordinates were obtained from dengue cases' households. Study site administrative shapefile boundaries were freely downloaded from National Bureau of Statistics website and used to create a map layer.

### Ethical considerations

Volunteers were recruited on the written informed consent form. Ethical approval was granted by Ifakara Health Institute (IHI) (IHI/IRB/No: 27–2014) and the National Institute of Medical Research (NIMR/HQ/R.8a/Vol. IX/1866).

## Results

### Demographic features of confirmed dengue cases

The average age of dengue cases surveyed was 32 years. There was a wide age range among cases with the oldest being 97 years old. Dengue cases of less than 18 years were not involved due to ethical reasons even though there were around 30 confirmed cases of dengue among children (Emiliana Athumani, pers comm). Therefore, descriptions refer to adult cases only and may not be generalizable to children. Slightly more males than females were hospitalised with dengue 57% (95% CI 46.7–66.7) against 43% (95% CI 33.3–53.3), (Table 2). The median time between patient diagnosis and larval sampling was 13 (IQR 7–16 weeks).

**Table 2:** Summary of patients with dengue fever (n = 97).

Variable	Categories	n	(%)	[95% CI
Gender	Male	55	57	[47, 67]
	Female	42	43	[33, 53]
Age categories	18–24	31	32	[21, 41]
	25–34	35	36	[26, 46]
	35–50	22	23	[14, 31]
	51 +	9	9	[03, 15]
Means of health education messaging accessible by the population	Mobile phone	96	99	[96, 100]
	Radio	77	79	[71, 88]
	Television	75	77	[69, 86]

**Fig 3:**
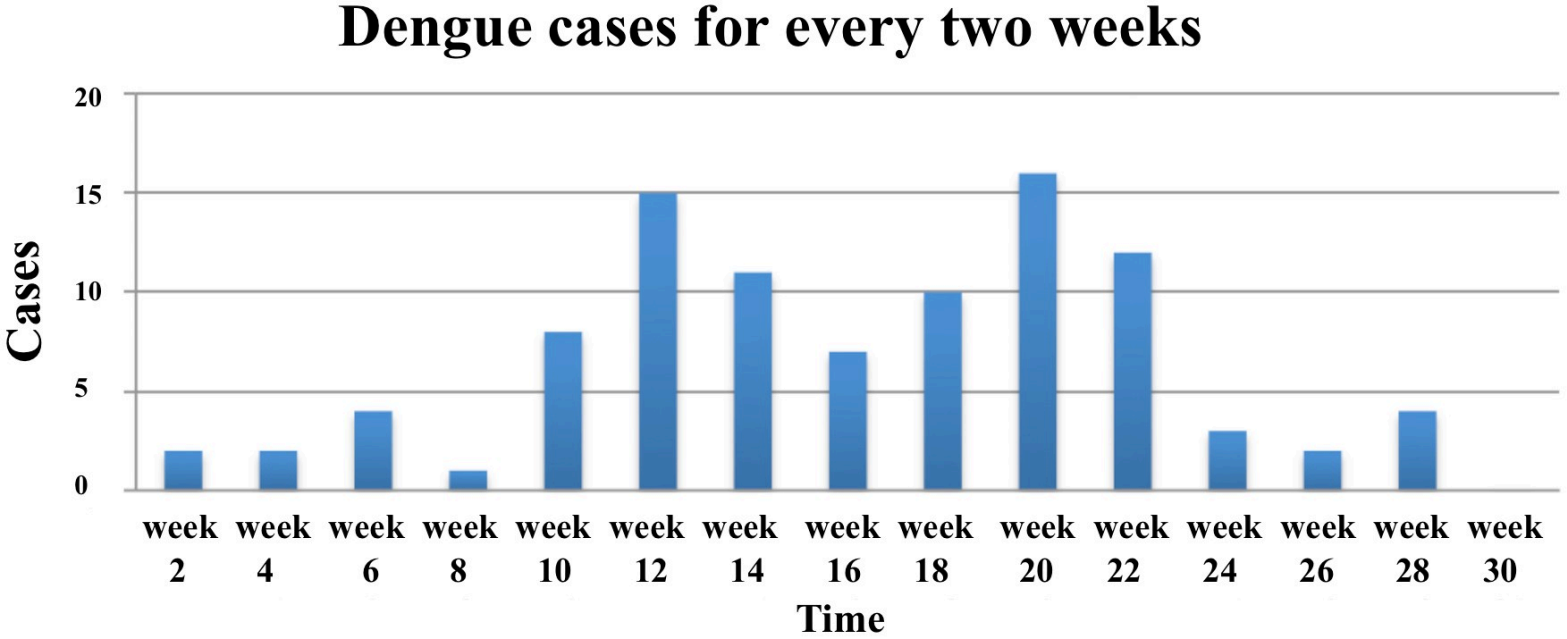
Fortnightly accrual of confirmed dengue outbreak cases at Mwananyamala hospital clinic in Dar es Salaam Tanzania between January and July 2014 (n = 97).

The accumulation of dengue cases suggests a propagated outbreak of dengue virus in the population. This can be seen by a steep increase of dengue cases and incremental jumps. The jumps reflect the generation of new cases in the population “Fig 3”. The two peaks were in April and July. Dengue cases density was higher in the wards that had market places in the Kinondoni municipality (Fig 2). However, distances from the markets to dengue cases houses were not measured. Manzese ward had the highest number of cases 18, followed by Mwananymala, Tandale and Mabibo that had 13, 13, and 9 cases, respectively.

**Fig 4:**
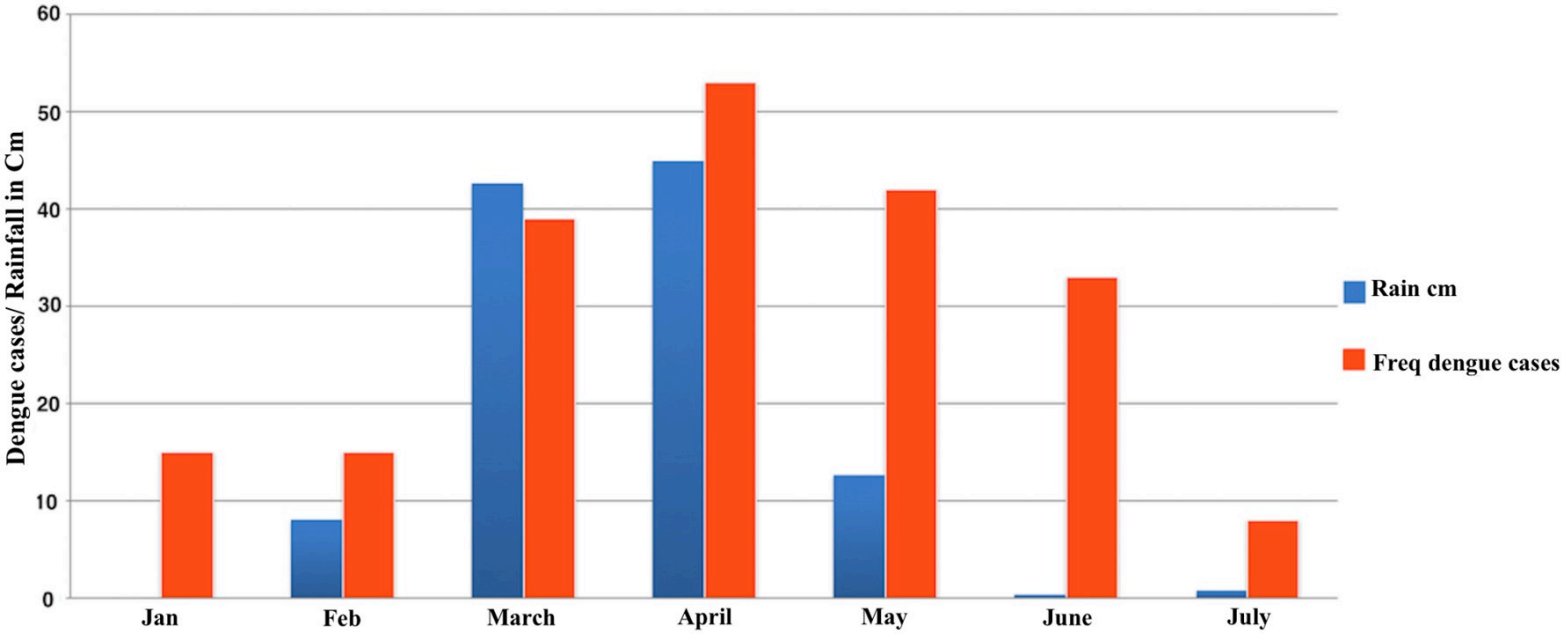
The relationship between rainfall and dengue outbreak cases.

The analysis of rainfall and dengue cases involved the incidences of 202 cases confirmed in Kinondoni district “Fig 4”. There is a monotonic relationship between rainfall and dengue cases with a Spearman’s correlation of 0.69 (p = 0.05).

### Potential breeding habitats

**Fig 5:**
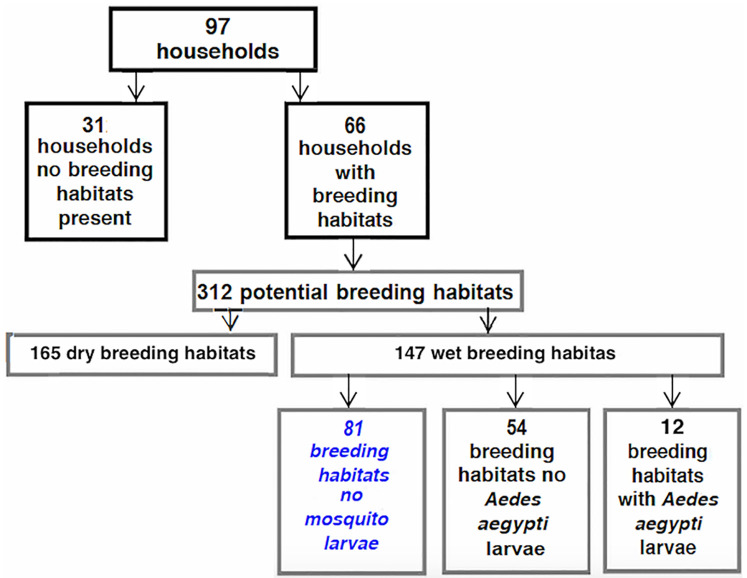
Distributions of PBHs across households and productive breeding habitats.

There were 312 PBHs surveyed from 66 households “Table 3” and 31 households did not have any visible breeding sites. Aedes larvae were found in only 4% (12/312) breeding habitats among 12 different households “Fig 5”. All the *Aedes* hatched *were Ae*. *aegypti* and no other potential arbovirus vectors such as *Ae*. *africanus* and *Ae*. *albopictus* were found.

**Table 3:** Frequency of common Aedes *aegypti* breeding habitat types across households.

Breeding habitat type	Total number	Location		Number of Households *PBH observed	(%)
		Outdoor	Indoors		
Drums/Barrels	43	17	26	28	18.8
Flower pots	87	58	29	26	17.4
Garbage	31	31	0	18	12.1
Plastic bags with accumulated water	54	54	0	14	9.4
Tires	56	48	8	25	16.8
Ground pool	13	13	0	12	8.1
Sandpit	4	4	0	3	2.0
Pond	3	3	0	1	0.7
Water collection	21	21	0	22	14.8
	312	249	63		

* Potential Breeding Habitat

Most of the PBHs were located outdoors. The most common PBHs were flowerpots followed by tyres and plastic bags with accumulated water “Table 3”. Drums and barrels, which are usually kept indoors, on verandas and corridors used for water storage were also common and found in the highest proportion of households (18.8%).

### Habitat Suitability Score (HSS)

**Fig 6:**
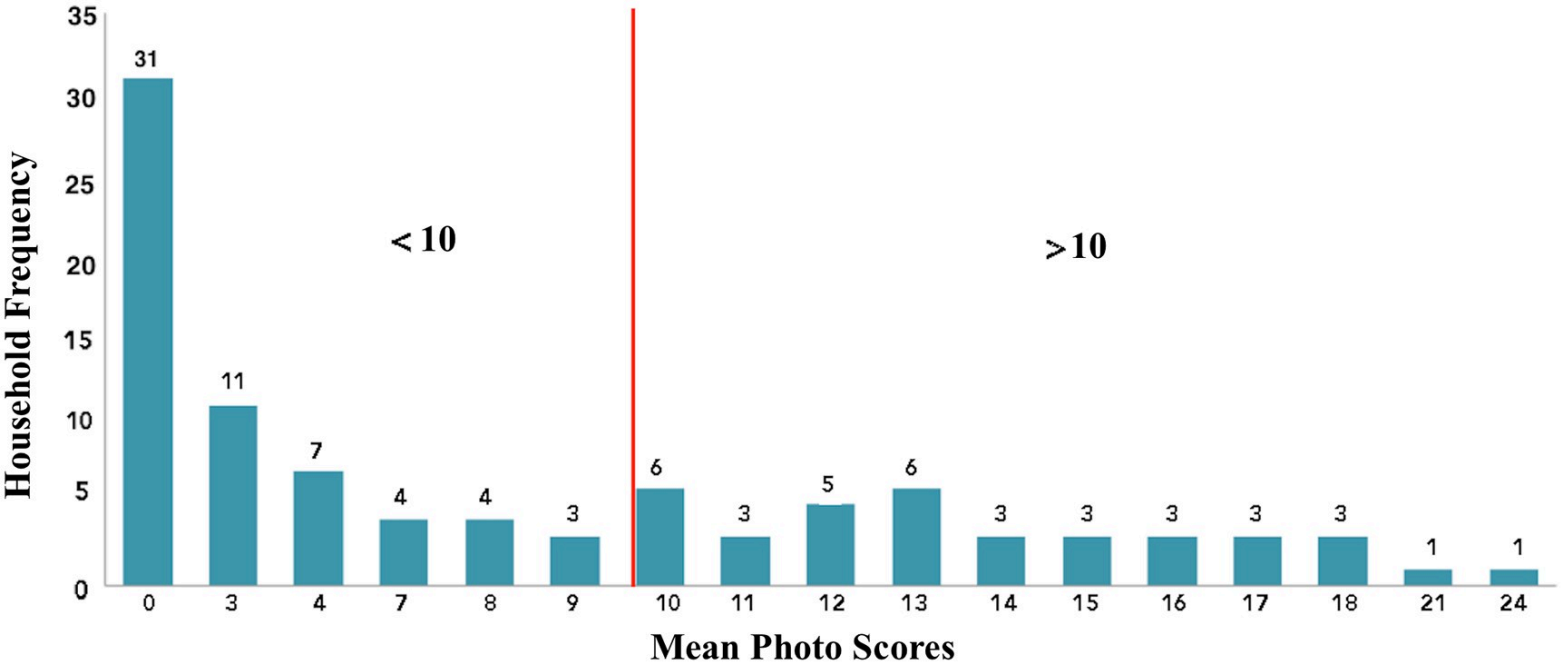
The habitat suitability scores distribution across households with dengue cases.

The highest household HSS was 24 points and the lowest had 3 points. Mean score was 10.8 points “Fig 6”. Two steps were used to validate HSS. Firstly, the assessment included all photo-scores and in absence Aedes and other mosquito larvae (Table 1). Secondly, the photoscore of the same habitats but at this time it included Aedes larvae scores after laboratory results of hatched larvae. A two-tailed paired t-test was used to assess the means difference between the two samples. There was no evidence of a difference between the two means, TTest = 1.13, p-value 0.26. HSS correctly predicted 9/12 breeding habitats positive for *Ae*. *aegypti* larvae in the dry season out of the twenty highest ranked PBHs by scores. It also identified that 37 out of 97 households contributed the majority of the breeding sites in the dry season and that these houses had a HSS > 10. Therefore, using the HSS could reduce the number of houses visited for larval source reduction by 2/3 and still include most of PBHs “Fig 6”.

### Dengue sign and symptoms

Dengue patients were asked to list the signs and symptoms of dengue fever using an open-ended question. A total of 73 (75%) of them mentioned fever and 82 (85%) mentioned a headache. Other signs and symptoms were: body malaise 62 (63%), joint pain (62%), muscle pain, 26 (27%), eye pain 20 (21%) and vomiting 15 (16%). Other signs and symptoms seldom mentioned were excessive sweating, epistaxis, abdominal pain and diarrhoea.

### Dengue awareness

When asked about where they got information about dengue fever many of the respondents (60%) said at the hospital (Table 4). The majority of dengue patients only became aware of dengue fever when they were already infected. Despite a widespread radio, newspaper and television coverage of the epidemic, radio and television were only mentioned as a source of information about dengue by 38% and 25% of respondents, respectively, although there was higher radio ownership among dengue patients’ households 79%. None of the respondents mentioned attending a local government meeting about dengue infection during the outbreak while seeing banners and public announcements were mentioned by 1% of respondents recalling dengue fever information.

**Table 4:** Knowledge and practice among confirmed dengue patients from the 2014 dengue outbreak in Dar es Salaam, Tanzania (n = 97) and multiple responses were allowed.

	Variable		n	%	[95% CI]
Where did you travel to in the past two weeks before you had dengue fever?	Did not Travel		77	79.38	[71, 86]
	Travelled Tanzania		16	16.49	[09, 24]
	Travelled Overseas		4	4.12	[00, 08]
Where did you hear about dengue?	Radio		37	38%	[28, 47]
	Television		25	25%	[17, 35]
	Brochure		6	6%	[01, 11]
	Public Announcement		1	1%	[-01, 03]
	Hospital		59	60%	[52, 71]
How do you protect yourself from dengue mosquito bites?	Use of Bednet		84	86%	[79, 93]
	Long dress &trousers		16	16%	[08, 24]
	Mosquito mesh		26	27%	[17, 35]
	Aerosol Spray		43	44%	[34, 54]
	Mosquito coil		6	6%	[01, 11]
	Cut grass outdoors		2	2%	[-01, 05]
	Fan		20	21%	[12, 29]
	Topical repellent		12	12%	[06, 19]
What are the signs and symptoms of dengue?	Fever	Yes	73	75	[67, 84]
		No	24	25	[16, 34]
	Headache	Yes	82	85	[77, 92]
		No	15	15	[08, 23]
	Joint pains	Yes	60	62	[52, 72]
		No	37	38	[28, 48]
	Vomiting	Yes	15	16	[08, 23]
		No	82	84	[77, 92]
	Eye pain	Yes	20	21	[12, 29]
		No	77	79	[71, 86]
	Muscle pain	Yes	26	27	[18, 36]
		No	71	73	[64, 82]
	Bleeding	Yes	8	8	[03, 14]
		No	89	92	[86, 97]
Body Malaise	Yes		62	63	[52, 74]
	No		35	37	[26, 47]
How is dengue transmitted	Person to person		21	22	[12, 29]
	Mosquito		67	69	[52, 76]
	Contaminated water		2	2	[-02, 06]
	Don’t Know		17	18	[10, 26]

Even though all participants had gone to the hospital and been diagnosed with dengue fever they remained poorly informed about the route of dengue transmission. Although 69% of respondents did mention that mosquitoes transmit the disease, 22% of respondents reported that dengue fever is spread from one person to another, 2% thought that dengue fever is spread by drinking water and 17% did not know how dengue fever is spread.

### Travel history

A total of 72 (70%) dengue patients had not travelled out of Dar es Salaam suggesting autochthonous transmission. Of those who travelled, the majority went to other parts of Tanzania, Africa and Asia; 24%, 2%, and 2%, respectively. Two of the dengue patients recorded at Mwananyamala hospital were not Dar es Salaam residents but they came to Dar es Salaam during the dengue outbreak. They got sick while in Dar and were treated in Kinondoni District.

## Discussion

Understanding the pattern of dengue fever and risk factors associated with transmission in Dar es Salaam it is of great significance to prevent future outbreaks through targeted control of dengue vectors and disseminating appropriate behaviour change communication messages to 1) improve household level environmental modification or destruction of breeding sites around homes, 2) to encourage adherence to personal protection and 3) identification of disease symptoms of the disease as is successfully used in many endemic countries [25]. Future outbreaks have a high probability of occurring more frequently since there have been outbreaks in 2007–8[8], 2010 (Type III detected in Zanzibar)[26], 2013 (Type II)[27] and 2014 (Type II)[6] as well as locally reported cases in 2017 and 2019[28]. However, knowledge among communities and about non-malaria febrile illnesses is extremely low and when attending health services, clinical diagnosis for febrile illnesses lacks specificity and has been reported to contribute to misdiagnosis and mistreatment of febrile patients [29].

The HSS is proposed by this study to be used as a tool for dengue mitigation before rains begin. During the dry season, the tool was able to correctly identify 75% of breeding habitats that were positive for *Ae*. *aegypti*. The fact that only a few breeding sites produce the most mosquitoes means that the tool may help guide the effect use of limited resources for larval source management. The tool can either be used by assessing photographs of PBHs or used directly on-site as a surveillance tool. It is an easy to use tool that doesn’t require formal training but to follow instructions to score points based on what the user sees. It is a tool planned to be used by house owners for assessing Aedes breeding areas in their premises. This will eventually bring vector control methods to the household level. This tool is only reported here and needs further validation in Dar es Salaam and other sites with *Ae*. *aegypti* to measure its efficiency for predicting suitable habitats and areas where targeted environmental management should be conducted.

Erratic water supply may have led to many households have drums, barrels for water storage while house decorations led to an abundance of flowerpots. Improper disposal used tires may have led to a rampant scattering of used car tyres. All these notoriously common sources of Aedes breeding as it was also found by Lutomiah *et al*[30] and Ngugi in Mombasa[31] as well as Mboera and colleagues in Dar es Salaam [6] who identified large items of household waste such as buckets and tyres to be the most important sources of mosquitoes breeding areas. Numerous outdoor breeding habitats that retain rainwater for long enough to allow Aedes to lay eggs and hatch and should be assessed for their productivity.

**Fig 7:**
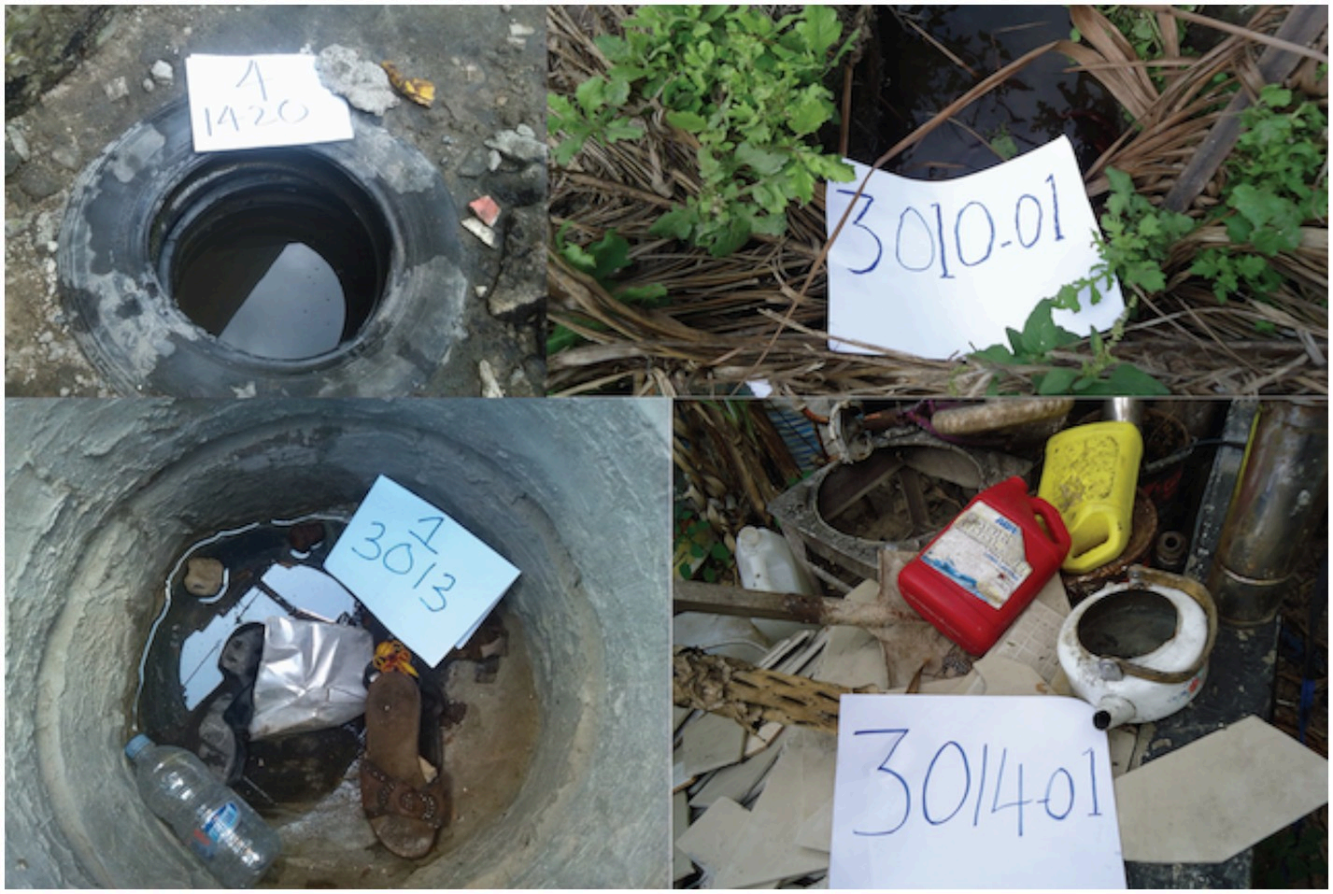
Some of the breeding habitat types found around households that had positive dengue cases traced from Mwananyamala hospital during the 2014 dengue outbreak (n = 97).

The rainy season amplifies the number of Aedes mosquitoes and unusually heavy rains during climate anomalies such as El Nino are often associated with outbreaks of dengue [32]. The increase in available breeding sites and consequently of the vectors is likely to have amplified dengue infection which was likely to be subclinical in a sufficient number of people to turn into an outbreak [33]. Nonetheless, dengue cases declined as the rainy season came to an end in July. This may also suggest that outdoor breeding habitats “Fig 7” were responsible for increasing densities of dengue vectors and may have facilitated the spread of dengue virus as has also been seen in neighbouring Kenya [31]. Furthermore, we have seen that only 4% of breeding habitats inspected were positive with dengue larvae, it may suggest that with favourable conditions just a small vector foci is adequate to cause an outbreak.

### Effects on pre-existing immunity

The 2014 outbreak did not have a lot in common with previous outbreaks and in common with most of recent past outbreaks did not have baseline data [34]. Severe dengue was not common [17]. Cases were few and many individuals sought treatment at a private clinic such as the International School of Tanganyika (IST) clinic in Dar es Salaam [35].

It was noted in 2014 that individuals who had more than one infection with dengue had severe outcomes and took a long time to recover [35]. Furthermore, people of 15 years and above were more affected and most cases occurred in Kinondoni municipality. This severity was probably due to a new strain of dengue identified to be DENV2 in 2014. The earlier outbreak strain in the country was DENV3[34–36].

There were patterns of more dengue cases in wards that had market places “Fig 2” a further study will need to look into this. At the moment it will be treated as a suggestive of improper refuse disposal that may have a role to play in the spread of dengue in such areas. Therefore environmental control and public awareness/education about breeding habitats before the rain begins should be prioritized. None of the Aedes found was *Ae*. *africanus*, which maintains sylvatic, yellow fever, or *Ae*. *albopictus* that is an introduced southeast Asian species, competent for dengue that shares breeding sites with *Ae*. *aegypti* currently invading into several African countries [37] but has not yet been found in Tanzania[6, 38]

### Dengue public awareness

Many of the interviewed 60%, learned about dengue only when they were already sick. Although dengue is a neglected disease, the outbreak lasted for over six months. It is recommended that efforts are made to allow people to receive information at the outset of outbreaks of dengue so that they may contribute to proactive elimination of vector breeding sites, use personal protection and seek treatment early.

Using radios to convey disease awareness may have not been a successful means to inform the population simply because there was a lower recall of radio information about dengue fever (Table 4). Households’ access to radio was found to be 77% however information about dengue was reported by 38% of the patients. This is almost half of those who have radios. This could be that there were infrequent radio broadcasts and also the time of broadcasting could have missed those at work. Furthermore, television broadcast is only likely to reach the richer population. It is therefore suggested that messages to raise awareness for future outbreaks should be specifically tailored to specific groups by specific medium [39]. Almost all households with dengue patients had at least one mobile phone “Table 3”. Therefore, they provide a better platform to disseminate outbreak information to the public. In recent years mobile banking and other financial services through mobile phones have led to the exponential increase of mobile phone ownership which is at 73% in Tanzania [40]. A public health tailored message about an outbreak may efficiently be disseminated through mobile phones [41] as is currently being developed for community control and education in India.

### Study limitations

This was an observational study in which we were not in control of most variables and we had to rely on information already obtained of dengue patient at the dengue clinic. The clinic was a designated hospital for all dengue patients in the whole of Kinondoni District. The study took place towards the end of both the dengue outbreak and the rainy season, which in practice limited the study to ascertain real-time relationships between cases, vectors and PBHs. Some social-environmental variables had changed, which included PBHs, removal of some waste as well as fumigation, which reduced dengue vectors.

Areas that were examined for breeding habitat were not uniform and surface area not measured. The area around households varied leading to some households having more breeding habitats. The study neither standardised the surveyed areas of PBH nor set a specified quadrant for the survey.

We also cannot establish temporal relationships apart from relying on two weeks of dengue fever incubation period in which we assumed that patients came to the clinic in just a day or two after the onset of the disease. There could also be recall bias from some respondents who were sick several months before we collected information. The study may also have missed some dengue cases that had insufficient discomfort to require medical attention.

There was low reporting of dengue information from radio or television, the study did not ask about the time of broadcasting dengue information from neither broadcaster nor patients. The broadcasting time could result in a lower number of listeners if it was during work hours. This is a descriptive observational study, which makes the results mainly suggestive for future studies and not conclusive concerning risk factors.

### Conclusion

Because dengue outbreaks in Tanzania are not spontaneous but rather related to the rainy season, it is, therefore, possible to counteract dengue outbreaks by using proactive mitigations before and during the rains. A simple tool such as HSS may be used by to inform vector control teams and communities even when there are no rains to identify PBHs, which can be removed or covered to prevent the breeding of vectors. Such tools with further testing and improvements may become very useful for the control of arboviral infections at the household level and require no special skills. On public awareness and preventive measure, the study advocates the use of mobile phones to disseminate accurate and timely health information. The thriving access of mobile phone ownership and uses should be used to deliver speedy information about dengue preparedness and prevention by utilising hotlines and tailored text messages. This had not been part of the government vector control strategy which may benefit from a shift from reactive to proactive vector control [42].

Mass education on vector breeding habitats that involve the management of all containers at the compound that may hold water, frequent emptying and cleaning water-storage vessels, cleaning of gutters; covering stored tyres from rainfall, recycling or discarding of non-essential containers is a proven, low cost intervention and should become a focus for the environmental dengue prevention.

Dengue preparedness information to health care providers just before the rainy seasons will help to detect initial cases of dengue before they turn into an outbreak. All patients who present with classical signs and symptoms need to be provided with information on how to clear breeding sites from their compounds to prevent further perpetuation of the virus.

## Supporting information

S1 File VRC06Dengue_Fever_Household_Epid_Questionnaire. (PDF)

S2 File VRC06Dengue_Fever_Breeding-site-field_form_Questionnaire. (PDF)

S1 Checklist STROBE checklist. (DOCX)

S1 Data Data repository: http://data.ihi.or.tz/index.php/catalog/264. (DOCX)
