# Supplementary material for: Knowledge, attitudes and bite prevention practices and estimation of productivity of vector breeding sites using a Habitat Suitability Score (HSS) among households with confirmed dengue in the 2014 outbreak in Dar es Salaam, Tanzania
Source: PLoS Negl Trop Dis. 2020 Jul 2;14(7):e0007278. doi: 10.1371/journal.pntd.0007278 (PMC7363105; doi:10.1371/journal.pntd.0007278)
Supplement: S1 File — (PDF) [file pntd.0007278.s001.pdf]

HOUSEHOLD QUESTIONNAIRE

Introduction: Hello, my name is "………..". I am from IHI and work on a project investigating where Dengue is found in Dar es Salaam. We would like to ask you some questions.

To be filled in before the interview

0.1 Code of interviewer |____|____|

0.2 Date of interview |____|____| / |____|____| / |____|____|____|____| (Day/Month/Year)

0.3 Name of district |____|

0.4 Name of ward |____|

0.6 GPS coordinates of household: S: |__|__|__|__|__|__|__| E |__|__|__|__|__|__|__|

0.7 Did you or a member of your household recently have a case of Dengue?	[__] Yes<br>[__] No – STOP
0.8 INFORMED CONSENT OBTAINED:	[__] Yes<br>[__] No – STOP

Section 1: Household characteristics

"Now I would like to ask you some general questions about this household."

Q #	Questions and filters	Coding category	Answer (enter coding categories)
1.1	Who is responding to the questions?	01…Head of household<br>02…Partner of household head<br>03…Other adult in household, specify	|__|__| ____________________
1.2	How old is the respondent?	***Age in years***	|__|__| if less than 18, STOP

1.3	What is the main source of income in the household head?	01…Salary<br>02…Business<br>03…Farming/livestock keeping<br>04…Skilled labour (fundi)<br>05…Casual labour (kibarua)<br>06…Fishing<br>07…Driver/taxi/bajaji<br>08…Student<br>09…Pension<br>10…No source of income<br>11…Other, specify	|__|__| <br>______________________
1.4	What is the main material of the roof?<br>***Observe***	01…Grass /palm thatch<br>02…Corrugated iron sheets<br>03… Other metal, e.g. korie<br>04… Other, specify	|__|__| <br>______________________
1.5	What is the main material of the walls?<br>***Observe***	01…Mud and sticks<br>02…Burned bricks<br>03…Cement bricks<br>04…Mud bricks (Matofali mabichi)<br>05…Other, specify	|__|__| <br>______________________
1.6	What is the main material of the floor?<br>***Observe***	01…Earth<br>02…Cement<br>03…Tiles<br>04…Wood<br>05…Other, specify	|__|__| <br>______________________
1.7	Are any of the windows screened with netting?<br>***Observe***	01…Yes<br>00…No, **go to 1.8**	|__|__|
1.7a	What are the windows screened with?<br>***Observe***	01…Wire mesh (metal/plastic)<br>02…Old bednet<br>03…Other material, specify	|__|__| <br>______________________
1.8	Does the house have an open eave gap? ***Observe***	01…Yes<br>00…No	|__|__|
1.9	What is the main type of ceiling?<br>***Observe***	01…No ceiling<br>02…Wooden boards (unfitted)<br>03…Wooden plates or fitted boards<br>04…Gypsum<br>05…Traditional e.g. leaves, mud<br>06…Other, specify	|__|__| <br>______________________

1.10	What type of fuel does your household mainly use for cooking?	01…Electricity<br>02…Gas<br>03… Kerosene<br>04… Diesel-powered generator<br>05…Charcoal<br>06…Firewood/straw<br>07…Other, specify		|__|__| <br>________________________
1.11	Does your house use any of the following sources of energy?<br><br>***Prompt each category.***	01…Yes<br>00…No	Electricity<br>Hurricane lamp<br>Candle<br>Traditional lamp<br>Fire<br>Battery/solar<br>Generator<br>None<br>Other, specify	|__|__| |__|__| |__|__| |__|__| |__|__| |__|__| |__|__| |__|__| __________________________
1.12	What is the principal type of toilet facility used by members of the household?	01…Own flush toilet<br>02…Shared flush toilet<br>03…Own pit latrine<br>04…Shared pit latrine<br>05…Bush/forest/field		|__|__|
1.13	What is the principal household source of drinking-water?	01…Piped water in home or yard/bottled water<br>02…Rain water collection<br>03…Own well/pump<br>04…Shared well/pump<br>05…River/stream/pond/lake<br>06…Water truck/cart<br>07…Other, specify		|__|__| <br>__________________________
1.14	Does your household possess any of the following items?<br><br>***Prompt each category***	01…Yes<br>00…No	Mobile phone<br>Radio<br>Refrigerator/freezer<br>Electric Fan<br>Television<br>Satellite Dish/Cable<br>Generator<br>Air conditioner<br>None of the above	|__|__| |__|__| |__|__| |__|__| |__|__| |__|__| |__|__| |__|__| |__|__|

1.15	Does the household (any member) have any of the following means of transport?<br><br>***Prompt each category***	01…Yes<br>00…No	Bicycle<br>Motorbike<br>Car/Truck<br>Bajaji<br>Animal/Cart<br>Boat/Canoe/Dhow<br>None of the above	|__|__| |__|__| |__|__| |__|__| |__|__| |__|__| |__|__|
1.16	Does the household have any of the following close to the house?<br><br>***Observe a 20metre radius around the house do not ask***	01…Yes<br>00…No	Water storage jars<br>Plant pots<br>Discarded containers<br>Plastic bags<br>Car tyres<br>Pools of water<br>None of the above	|__|__| |__|__| |__|__| |__|__| |__|__| |__|__| |__|__|
**Section 2: I would like to ask you some questions about your past travel and work place**				
2.1	Where did you travel to in the past two weeks before you had dengue fever?<br><br>***Prompt each category***	01…Yes<br>00…No	Did Not travel<br>Up country<br>East Africa<br>Other Africa<br>Middle East/ Arab<br>Asia<br>America<br>South America<br>Australia/Oceania	|__|__| |__|__| |__|__| |__|__| |__|__| |__|__| |__|__| |__|__| |__|__|
2.2	Do you commute to your working place/ business?<br><br>***Prompt if working place is area near home which doesn't require commuting***	01…Yes<br>00…No	Do not travel<br>Once a day<br>Frequently in a day<br>Weekly<br>Monthly	|__|__| |__|__| |__|__| |__|__| |__|__|
**Section 3: "*Now I would like to ask you some questions about dengue.*"**				
3.1	How is dengue spread?<br><br>***Do not Prompt but discuss and check each answer you***	01…Yes<br>00…No	Human-human<br>Mosquito bite<br>Don't know	|__|__| |__|__| |__|__|

	hear mentioned		Other	|__|__| ________________________
3.2	How can you stop dengue from spreading?<br>***Do not Prompt but discuss and check each answer you hear mentioned***	01…Yes<br>00…No	Control mosquitoes / stop mosquito bites<br>Take medicine	|__|__| |__|__| |__|__| |__|__| |__|__| |__|__| |__|__|
3.2	What are the symptoms of dengue?<br>***Prompt each category***	01…Yes<br>00…No	Fever<br>Headache<br>Joints hurt<br>Feeling sick/vomiting<br>Don't know<br>Other	|__|__| |__|__| |__|__| |__|__| |__|__| |__|__| ________________________
**Section 4: "*Now I would like to ask you some questions about your bednets and mosquito control.*"**				
4.1	How many sleeping places are there in your household?<br>***Include all sleeping spaces where a net could be hung up, or has ever been hung up, including if there is more than one sleeping space in each room used for sleeping***			Indoors |__|__| Outdoors |__|__| Indoors Temporary |__|__| Outdoors Temporary |__|__|
4.1a	How many sleeping places <u>were used</u> last night in your household?			Indoors |__|__| Outdoors |__|__| Indoors Temporary |__|__| Outdoors Temporary |__|__|
4.2	How many mosquito nets that can be used for sleeping does your household have in total?<br>***Probe for nets not in use: stored, saved, unopened***			|__|__|
4.3	Which methods do you use to stop Dengue mosquitoes biting you?<br>***Do not Prompt but discuss and check each answer you hear mentioned***	01…Yes<br>00…No		Bednet |__|__| Long clothes |__|__| Screen house |__|__| Spray insecticide |__|__| Mosquito coil |__|__|

			Clean vegetation around house |__|__| Use a fan |__|__| Use an electricutor |__|__| Mosquito repellant cream/lotion |__|__| Empty water from trash or containers around the house |__|__|
4.4	Where do you use these methods?	00…Indoors<br>01…Outdoors<br>02…Indoors and outdoors	Bednet |__|__| Long clothes |__|__| Screen house |__|__| Spray insecticide |__|__| Mosquito coil |__|__| Use a fan |__|__| Use an electricutor |__|__| Mosquito repellant cream/lotion |__|__|
4.5	What time of day do you normally use these methods to stop mosquito bites?	00…Evening<br>01…Night<br>02…Evening and night	Bednet |__|__| Long clothes |__|__| Screen house |__|__| Spray insecticide |__|__| Mosquito coil |__|__| Use a fan |__|__| Use an electricutor |__|__| Mosquito repellant cream/lotion |__|__|
4.6	How often do you normally use these methods to stop mosquito bites?	00…Daily<br>01…Several times a week<br>02…About one time a week<br>03…Less than one time a week<br>04…When mosquitoes are a nuisance<br>91…Don't Know/ Don' t use	Bednet |__|__| Long clothes |__|__| Screen house |__|__| Spray insecticide |__|__| Mosquito coil |__|__| Use a fan |__|__| Use an electricutor |__|__| Mosquito repellant cream/lotion |__|__| Clean vegetation around house |__|__| Empty water from trash or containers around the house |__|__|

4.7	What do you (or the person who had dengue if under 18) do in the evenings after about 4pm until you go to bed?<br><br>***Try to talk about this and get some idea of the times when these activities are taking place.***<br><br>***Write the time that the person is doing each activity roughly***		At work |__|__|-|__|__| Coming home from work<br>|__|__|-|__|__| Visit friends |__|__|-|__|__| Go to a bar / restaurant<br>|__|__|-|__|__| Activities inside the house<br>|__|__|-|__|__| Sit outside the house<br>|__|__|-|__|__| Go to bed |__|__|-|__|__|
