# Supplementary material for: Knowledge, attitudes and bite prevention practices and estimation of productivity of vector breeding sites using a Habitat Suitability Score (HSS) among households with confirmed dengue in the 2014 outbreak in Dar es Salaam, Tanzania
Source: PLoS Negl Trop Dis. 2020 Jul 2;14(7):e0007278. doi: 10.1371/journal.pntd.0007278 (PMC7363105; doi:10.1371/journal.pntd.0007278)
Supplement: S2 File — (PDF) [file pntd.0007278.s002.pdf]

ROUTINE BREEDING SITE SURVEYS – FIELD FORM

DODOSO LA UKAGUZI WA MAZALIA

(TO BE FILLED IN FULL AT THE HOME VISIT)

(LIJAZWE KIKAMILIFU UNAPO ITEMBELEA KAYA)

Photo Number:/Namba ya picha |_______________| Sample serial/Namba |__|__|__|__|__|__|__|__|__|

K M M S S E E D D

Number of ward_Kata/Mtaa/South/East/Dip

0.0 Number of interviewee/ Namba ya mdodoswaji |__|__|__|__|

0.1 Code of interviewer/Namba ya mdodosaji |__|__|__|

0.2 Date of interview/Taraehe ya mahojiano |___|___| / |___|___| / |___|___|___|___| (Day/Month/Year)

0.3 Name of district/Jina la Wilaya |____|

0.4 Name of ward/Jina la Kata |____|

0.5 Street Name/ Jina la Mtaa |____|

0.6 GPS coordinates /Namba za GPS kaya ilipo: S: |__|__|__|__|__|__|__|__| E |__|__|__|__|__|__|__|__|

0.7 Je (Jina)………………ulipo/alipo ugua dengue ulikuwa unaishi hapa Ndiyo |__| Endelea na udodosaji
Hapana |__| Usiendelee,

Larval sampling/Sampuli ya viluilui:

0.1 Number of dips: Idadi ya mazalia/ Breeding Habitat |__|__|

0.2 Larvae present?/ Kiluilui yupo.

Culcine Y / N

Anopheines Y / N

Aedes Y/N

Habitat Type/Hali ya Mazingira (check if positive for *Aedes* larvae/ angalia uwepo wa viluilui wa Aedes)

Habitat		Habitat	
Dry / Pakavu		Discarded container/ Makopo yaliozagaa	
Ground pool/ Maji yaliyokusanywa		Plastic bag/ Mifuko ya Plastiki	
Stream / River margin/ Mfereji / kijito		Puddle / Hoof print/ Alama za kwato	
Agricultural overflow/ Mafuriko		Car tyre/ Matairi ya gari	
Water storage jar / bucket/ Ndoo na madumu		Brick or sand pit/ Mashimo ya udongo	
Plant pot / Makopo ya maua			

**Shade over habitat/ Kivuli juu ya mazalia**		**Habitat size: perimeter Ukubwa wa mazalia:mzingo**	
None/ Hakuna		Less than 10 cm/ Chini ya 10cm	
Partial/ Kwa kiasi		Between 10-100 cm/ Kati ya 10-100cm	
Heavy/Sana		More than 100 cm /Zaidi ya 100cm	

Water movement/ Mtiririko wa maji

Stagnant/ Yametuama	
Slow/ Taratibu	
Fast/ Kasi	

Water colour / Rangi ya maji

Clear/ Masafi	
Coloured/ Yana rangi rangi	
Polluted/ Machafu	

Water type: Aina ya Maji

Permanent/ Yapo sikuzote	
Semi-permanent/ Si ya kudumu	
Temporary/ Ya Muda	

Water depth/ Kina cha maji

Less than 5 cm/ Chini ya 5cm	
Between 5-15 cm/ Kati ya 5-15 cm	
More than 15 cm/ Zaidi ya 15 cm	

Environment (around water habitat) Eneo linalozunguka maji - **multiple ticks allowed / Yawezakuwa na jibu zaidi ya moja**

Scrub/Bush/ Kichaka	
Swamp/marsh: Tindiga	
Urban/ Eneo la Mjini	
Cultivated fields/ Shamba lililolimwa	

Distance from homes: Umbali toka nyumba ilipo

Less than 100 m/ Chini ya 100m	
Between 100-500 m/ Kati ya 100-500m	
More than 500 m/ Zaidi ya 500m	
