# Supplementary material for: Knowledge, attitudes and bite prevention practices and estimation of productivity of vector breeding sites using a Habitat Suitability Score (HSS) among households with confirmed dengue in the 2014 outbreak in Dar es Salaam, Tanzania
Source: PLoS Negl Trop Dis. 2020 Jul 2;14(7):e0007278. doi: 10.1371/journal.pntd.0007278 (PMC7363105; doi:10.1371/journal.pntd.0007278)
Supplement: S1 Checklist — (DOCX) [file pntd.0007278.s003.docx]

STROBE Statement—checklist of items that should be included in reports of observational studies

|  | Item No. | Recommendation | Page  No. | Relevant text from manuscript |
| --- | --- | --- | --- | --- |
| **Title and abstract** | 1 | (*a*) Indicate the study’s design with a commonly used term in the title or the abstract | Page 01. | Title |
|  |  | (*b*) Provide in the abstract an informative and balanced summary of what was done and what was found | Page 01 | Abstract |
| Introduction | | |  |  |
| Background/rationale | 2 | Explain the scientific background and rationale for the investigation being reported | Page 04 | Introduction  Paragraph 02 |
| Objectives | 3 | State specific objectives, including any prespecified hypotheses | Page 06 | Introduction  Paragraph 06 |
| Methods | | |  |  |
| Study design | 4 | Present key elements of study design early in the paper | Page 06 | Methodology Paragraph 01 |
| Setting | 5 | Describe the setting, locations, and relevant dates, including periods of recruitment, exposure, follow-up, and data collection | Page 06 | Methodology Paragraph 01 |
| Participants | 6 | (*a*) *Cohort study*—Give the eligibility criteria, and the sources and methods of selection of participants. Describe methods of follow-up  *Case-control study*—Give the eligibility criteria, and the sources and methods of case ascertainment and control selection. Give the rationale for the choice of cases and controls  *Cross-sectional study*—Give the eligibility criteria, and the sources and methods of selection of participants | Page 07 | Methodology Paragraph 02 |
|  |  | (*b*) *Cohort study*—For matched studies, give matching criteria and number of exposed and unexposed  *Case-control study*—For matched studies, give matching criteria and the number of controls per case | NA | NA |
| Variables | 7 | Clearly define all outcomes, exposures, predictors, potential confounders, and effect modifiers. Give diagnostic criteria, if applicable | NA | NA |
| Data sources/ measurement | 8* | For each variable of interest, give sources of data and details of methods of assessment (measurement). Describe comparability of assessment methods if there is more than one group | NA | NA  *All who were interviewed had an outcome of interest*. |
| Bias | 9 | Describe any efforts to address potential sources of bias | Page 07  Page 12 | **Methods**  Data collection  Paragraph 03  **Results**  Paragraph 04 |
| Study size | 10 | Explain how the study size was arrived at | Page 07 | Methods  Paragraph 02 |

| Quantitative variables | 11 | Explain how quantitative variables were handled in the analyses. If applicable, describe which groupings were chosen and why | NA |  | NA |
| --- | --- | --- | --- | --- | --- |
| Statistical methods | 12 | (*a*) Describe all statistical methods, including those used to control for confounding |  |  | It was a descriptive analysis did not involve multivariable analysis |
|  |  | (*b*) Describe any methods used to examine subgroups and interactions | NA |  | NA |
|  |  | (*c*) Explain how missing data were addressed | Page 07 |  | Methodology  Paragraph 03 |
|  |  | (*d*) *Cohort study*—If applicable, explain how loss to follow-up was addressed  *Case-control study*—If applicable, explain how matching of cases and controls was addressed  *Cross-sectional study*—If applicable, describe analytical methods taking account of sampling strategy | NA |  | Was a retrospective study |
|  |  | (*e*) Describe any sensitivity analyses | NA |  | NA |
| Results | | |  | |  |
| Participants | 13* | (a) Report numbers of individuals at each stage of study—eg numbers potentially eligible, examined for eligibility, confirmed eligible, included in the study, completing follow-up, and analysed | Page 11 |  | Results Paragraph 02 |
|  |  | (b) Give reasons for non-participation at each stage | NA |  | NA |
|  |  | (c) Consider use of a flow diagram | NA |  | NA |
| Descriptive data | 14* | (a) Give characteristics of study participants (eg demographic, clinical, social) and information on exposures and potential confounders | Page 12  Page 13 |  | Results  Paragraph 02  Also  Table 3 |
|  |  | (b) Indicate number of participants with missing data for each variable of interest | NA |  | NA |
|  |  | (c) *Cohort study*—Summarise follow-up time (eg, average and total amount) | NA |  | NA |
| Outcome data | 15* | *Cohort study*—Report numbers of outcome events or summary measures over time | NA |  | NA |
|  |  | *Case-control study—*Report numbers in each exposure category, or summary measures of exposure | NA |  | NA |
|  |  | *Cross-sectional study—*Report numbers of outcome events or summary measures | NA |  | NA |
| Main results | 16 | (*a*) Give unadjusted estimates and, if applicable, confounder-adjusted estimates and their precision (eg, 95% confidence interval). Make clear which confounders were adjusted for and why they were included | NA |  | NA |
|  |  | (*b*) Report category boundaries when continuous variables were categorized | Age category.  Page 13 |  | Results  Table 03 |
|  |  | (*c*) If relevant, consider translating estimates of relative risk into absolute risk for a meaningful time period | NA |  | NA |

| Other analyses | 17 | Report other analyses done—eg analyses of subgroups and interactions, and sensitivity analyses | Page 13 |  | Results  Paragraph 01 |
| --- | --- | --- | --- | --- | --- |
| Discussion | | |  | |  |
| Key results | 18 | Summarise key results with reference to study objectives | Page 17 |  | Discussion  Paragraph 02 |
| Limitations | 19 | Discuss limitations of the study, taking into account sources of potential bias or imprecision. Discuss both direction and magnitude of any potential bias | Page 20 |  | Discussion  Paragraph 01 |
| Interpretation | 20 | Give a cautious overall interpretation of results considering objectives, limitations, multiplicity of analyses, results from similar studies, and other relevant evidence | Page 20 |  | Discussion  Paragraph 01  Conclusion  Paragraph 01 |
| Generalisability | 21 | Discuss the generalisability (external validity) of the study results | NA |  | NA |
| Other information | |  |  | |  |
| Funding | 22 | Give the source of funding and the role of the funders for the present study and, if applicable, for the original study on which the present article is based | Page 21 |  | Acknowledgement  Paragraph 02 |

*Give information separately for cases and controls in case-control studies and, if applicable, for exposed and unexposed groups in cohort and cross-sectional studies.

**Note:** An Explanation and Elaboration article discusses each checklist item and gives methodological background and published examples of transparent reporting. The STROBE checklist is best used in conjunction with this article (freely available on the Web sites of PLoS Medicine at http://www.plosmedicine.org/, Annals of Internal Medicine at http://www.annals.org/, and Epidemiology at http://www.epidem.com/). Information on the STROBE Initiative is available at www.strobe-statement.org.
