# Supplementary material for: Knowledge, attitudes and bite prevention practices and estimation of productivity of vector breeding sites using a Habitat Suitability Score (HSS) among households with confirmed dengue in the 2014 outbreak in Dar es Salaam, Tanzania
Source: PLoS Negl Trop Dis. 2020 Jul 2;14(7):e0007278. doi: 10.1371/journal.pntd.0007278 (PMC7363105; doi:10.1371/journal.pntd.0007278)
Supplement: S1 Data — (DOCX) [file pntd.0007278.s004.docx]

[**http://data.ihi.or.tz/index.php/catalog/264**](http://data.ihi.or.tz/index.php/catalog/264)
